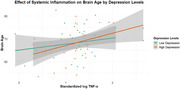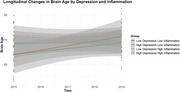# What matters more in brain aging associated with systemic inflammation: Depression or loneliness?

**DOI:** 10.1002/alz70860_101983

**Published:** 2025-12-23

**Authors:** Hyunjeong Kwak, Jeanyung Chey, Sangmin Park, Sung‐Ha Lee, Yoosik Youm

**Affiliations:** ^1^ Seoul National University, Seoul, Korea, Republic of (South); ^2^ Yonsei University, Seoul, Korea, Republic of (South)

## Abstract

**Background:**

Social isolation or loneliness is a risk factor for various health problems including dementia and depression (Kuiper et al., 2015; Donovan & Blazer, 2020). Depression is also known to be associated with cognitive decline (Evans et al., 2013; Byers & Yaffe, 2012). Loneliness and depression were associated with chronic low‐grade inflammation (Lee et al., 2023: Del Giudice & Gangestad, 2018), which can accelerate brain aging and increase dementia risk (Chey & Kwak, 2023). Few studies, however, have examined how these psychological factors influence the relationship between systemic inflammation and brain atrophy, especially longitudinally. This study investigated the longitudinal impact of these key negative psychological states on brain age, focusing on the inflammatory pathway.

**Method:**

Thirty healthy older adults (Mean Age=72.66, *SD* = 6.18; Male=14) from the Korean Social Life, Health, and Aging Project (KSHAP) study completed baseline and follow‐up assessments 4 years later. TNF‐α, pro‐inflammatory cytokine, was measured from blood samples and brain age was estimated using machine‐learning analysis based on structural MRI data. Participants completed Geriatric Depression Scale and UCLA Loneliness Scale. Linear mixed‐effects models examined whether the impact of systemic inflammation on brain age was affected by depression and/or loneliness.

**Result:**

A paired t‐test demonstrated significant increase in brain age and decreasing trend in depression over time. An independent sample t‐test suggested that changes in brain age marginally differed by levels of depression, with the higher depression group showing greater increase in brain age. Linear mixed‐effects analysis revealed that changes in TNF‐α significantly contributed to brain aging, with higher inflammation levels associated with greater increases in brain age. Moreover, a significant interaction was found between depressive symptom and changes in TNF‐α; i.e., systemic inflammation had a stronger effect on brain age in older adults with higher depression. In contrast, loneliness showed no significant effects in the LME analysis.

**Conclusion:**

The study found that depression, not loneliness, has a negative impact on brain aging associated with inflammaging over a four‐year period. Specifically, systemic inflammation accelerated brain aging only in older adults with significant depressive symptoms, suggesting the importance of alleviating depression as a key preventive strategy for cognitive decline or dementia in older adults.